# Detecting cellular reprogramming determinants by differential stability analysis of gene regulatory networks

**DOI:** 10.1186/1752-0509-7-140

**Published:** 2013-12-19

**Authors:** Isaac Crespo, Thanneer M Perumal, Wiktor Jurkowski, Antonio del Sol

**Affiliations:** 1 Luxembourg Centre for Systems Biomedicine (LCSB), University of Luxembourg, L-4362, Esch-Belval, Luxembourg

**Keywords:** Cellular reprogramming, Transdifferentiation, Dedifferentiation, Stability, Attractor, Positive circuit, Reprogramming determinants

## Abstract

**Background:**

Cellular differentiation and reprogramming are processes that are carefully orchestrated by the activation and repression of specific sets of genes. An increasing amount of experimental results show that despite the large number of genes participating in transcriptional programs of cellular phenotypes, only few key genes, which are coined here as reprogramming determinants, are required to be directly perturbed in order to induce cellular reprogramming. However, identification of reprogramming determinants still remains a combinatorial problem, and the state-of-art methods addressing this issue rests on exhaustive experimentation or prior knowledge to narrow down the list of candidates.

**Results:**

Here we present a computational method, without any preliminary selection of candidate genes, to identify reduced subsets of genes, which when perturbed can induce transitions between cellular phenotypes. The method relies on the expression profiles of two stable cellular phenotypes along with a topological analysis stability elements in the gene regulatory network that are necessary to cause this multi-stability. Since stable cellular phenotypes can be considered as attractors of gene regulatory networks, cell fate and cellular reprogramming involves transition between these attractors, and therefore current method searches for combinations of genes that are able to destabilize a specific initial attractor and stabilize the final one in response to the appropriate perturbations.

**Conclusions:**

The method presented here represents a useful framework to assist researchers in the field of cellular reprogramming to design experimental strategies with potential applications in the regenerative medicine and disease modelling.

## Background

During classical cellular differentiation cells lose phenotypic plasticity until they become fully differentiated. Some differentiated cells have the remarkable ability to be converted into different cell types via a process termed as developmental redirection or cellular reprogramming. Both differentiation and reprogramming are processes that are carefully orchestrated by the activation and repression of specific sets of genes. The knowledge about these activation and repression mechanisms can be integrated as network of regulations. Modeling these regulatory networks allow us to describe biological processes, in general, as transitions between network states and cellular reprogramming, in particular, as transitions between stable steady states also called as attractors of the network model. On the other hand, the relationship between cellular phenotypes and the attractors has been proposed by several authors [1-3], and recent literature authenticates this claim with experimental validation of a number of examples showing that only few key genes can induce transitions between cellular phenotypes [4-7].

Prediction of these key genes finds wide range of applications for cellular reprogramming. However, there is only handful of approaches in literature that can predict effective cocktails of transcription factors for cellular reprogramming [8,9]. Most of these methods either requires a list of candidate genes to narrow down the combinatorial problem or based on computational brute force to simulate network response under perturbation. Both the said strategies become prohibitive for the larger number of genes in the network. To this end, here we propose a computational methodology, which systematically identifies these key driver genes that are able to induce transitions between various cell types including differentiation, de/trans-differentiation.

Stable cellular phenotypes (representing attractors of our network model) are part of a large space of all available cellular states. At the transcriptional level, attractors represent stable expression patterns or transcriptional programs. The existence of multiple attractors in a GRN requires the presence of positive feedback loops or also called as positive circuits (i.e., including even number of inhibitions/repressive regulations) [10]. However, not all positive circuits in the network are involved in network multistability; those whose participating genes cannot be in a coherent stable state according to the connectivity of the circuit (i.e., mismatch between the logical rules and the expression pattern) are not contributing to stabilize the network because they are not stable by themselves. Moreover, there are positive circuits that are contributing to stabilize specific attractors but not another.

In a previously published work [11] we proposed the so called differentially expressed positive circuits (DEPCs) as targets to induce cellular transitions and showed how a topology based strategy pointed out genes involved in the so called bi-toggle switches (transcription factor cross-repressing motifs) as driver genes for these transitions. Here we used a bioinformatics approach to interrogate synthetic networks preserving properties of the well characterized gene regulatory network (GRN) of E. coli and we observed that there always exists at least one DEPC, which constitutes a necessary condition for the general applicability of the methodology presented here. A positive circuit is considered DEPC if its constitutive genes change their expression values between two given attractors of the GRN. Hence, we assume that DEPCs forms the barrier between the given two attractors. Therefore, appropriate perturbation of genes belonging to these differentially expressed stability elements is expected to destabilize the initial cellular phenotype and stabilize the final one.

Thus, by combining transcriptomics profiling, and stability analysis, proposed methodology identifies key genes, called here as reprogramming determinants (RDs), without considering any prior list of candidate genes. Here, RDs are defined as minimal set of genes, a single gene or group of genes, that are participating in the differential stability elements of the network model, when perturbed with an appropriate stimulus (either activation or repression) can effect transitions between stable cellular programs. In this formalism, there are no constraints on the nature of products encoded by RDs (i.e., key genes); both proteins as well as non-coding RNAs are equally eligible. Finally, RDs encompass as many number of gene combinations, as long as the set is minimal and can effect transitions between attractors of the network model.

The objective of this methodology is to identify all possible RDs which can bring about the cellular transitions. Here we propose a novel strategy to select combinations of genes to be perturbed based on dynamical simulations instead of the purely topology based strategy proposed before [11]. By focusing on genes involved on the stability of the gene regulatory network (GRN), the algorithm dramatically reduces the huge search space constituted by all possible combinations of genes. The efficiency and general usability of our methodology is demonstrated by analyzing a large number of *in silico* GRNs generated with biological properties as that of *E. coli* regulatory network, and selective six different biological examples of cellular reprogramming. Analysis of *in silico* gene regulatory networks showed that these minimal sets of driver genes were always able to trigger transitions between all pairs of attractors. Application to six biologically relevant examples finds experimental validation in literature for the identified sets of RDs as effective inducers of transitions between cellular phenotypes. Given the increasing interest of cellular reprogramming in regenerative medicine and basic research, our method represents a useful computational methodology to assist researchers in designing experimental strategies.

## Results

### Description of the differential expression stability analysis

**Figure 1:**
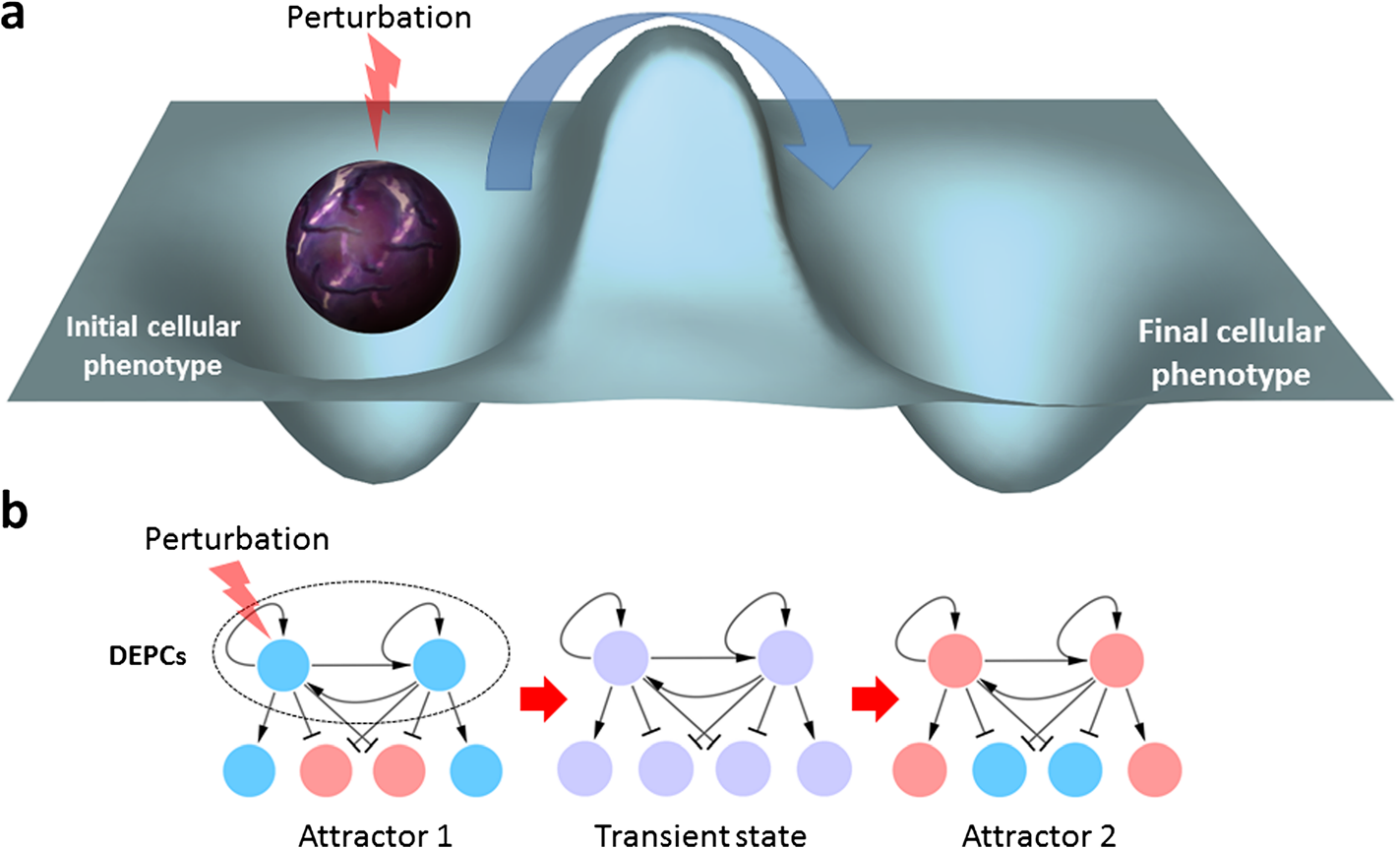
**Description of transitions between cellular phenotypes using transcriptional landscapes and networks. a)** Cell transcriptional program landscape representing two attractors and the epigenetic barrier between them. This conceptual figure represents a cell stabilized in an initial cellular phenotype and how a hypothetical perturbation can destabilize the cellular program and make cell exceed the barrier and fell down in a final cellular phenotype. This cellular reprogramming is represented as a blue arrow from the initial to the final attractor. **b)** Cellular reprogramming as transitions between network states. Differentially expressed positive circuits (DEPCs) are perturbed to induce the transition from Attractor 1 to Attractor 2 passing by a transient state. This transient state can be considered as a “short” term changing expression pattern until the system reaches an attractor. Regular arrows represent activation and T-arrows represent inhibitions. Blue and red nodes represent inactive and active genes respectively in attractors. Violet nodes represent transient states.

Cellular phenotypes are characterized by stable expression patterns at the transcriptional level. The underlying GRN can be conceptualized and described as Waddington landscape [12-14], where stable cellular phenotypes, corresponding to the attractors of network model, are represented as wells separated by barriers (see Figure 1). These barriers are established by those network elements that are stabilizing GRNs in their attractors. In the motive of identifying these barriers, the method presented here takes reconstructed GRNs and the associated expression patterns of the cellular phenotypes as input, and gives RDs as output. Since stable cellular phenotypes can be considered as attractors of GRNs, cell fate and cellular reprogramming involve transitions between these attractors. To this end, our method looks for combinations of genes in the reconstructed GRN that are able to destabilize a specific initial attractor and stabilize the final one in response to the appropriate perturbation. Therefore, this strategy allows us to narrow down a huge combinatorial searching problem to a set of minimal combinations that constitutes alternative reprogramming protocols. It is to note that this method operates on previously reconstructed GRNs (both from knowledge based or data based approaches).

**Figure 2:**
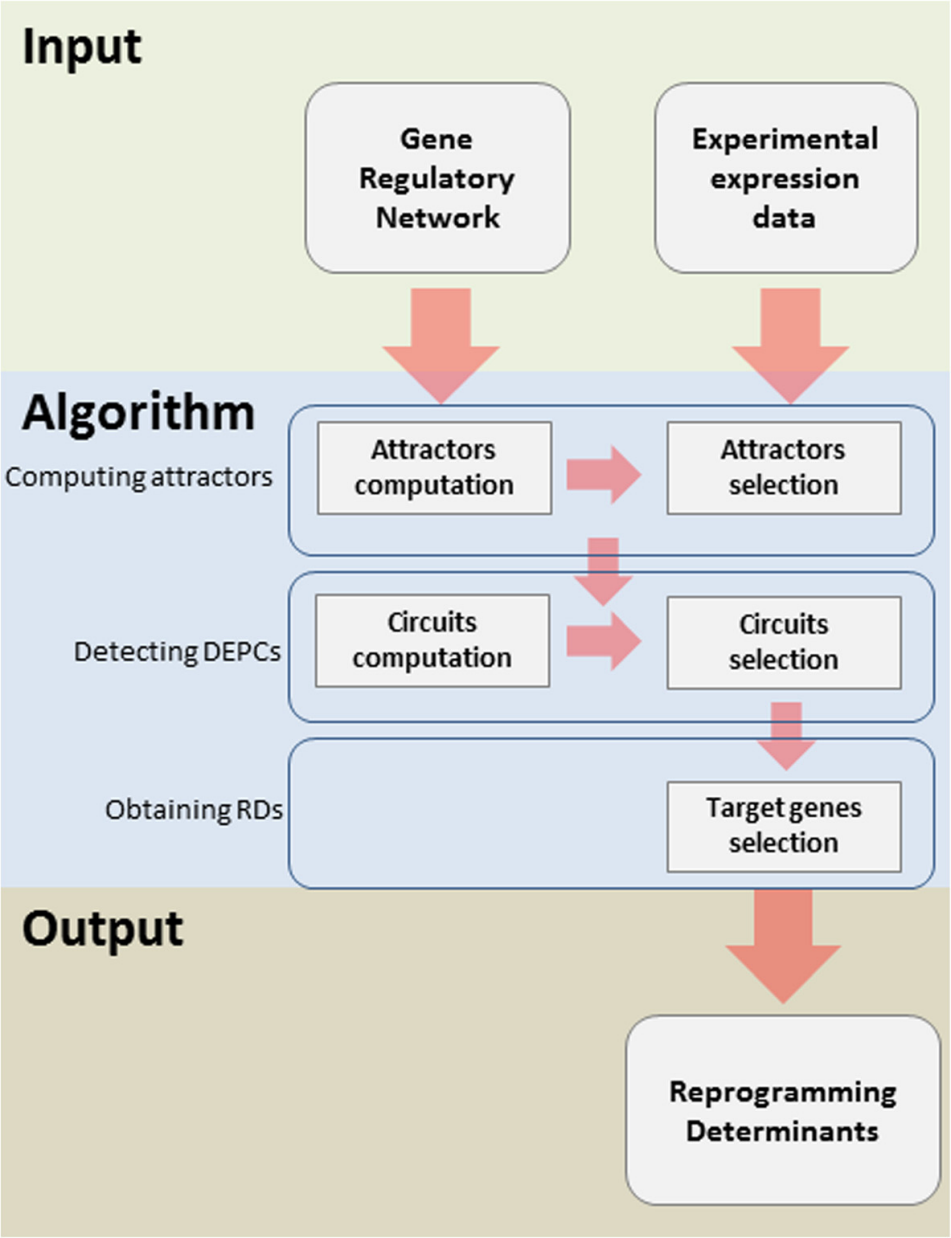
**Flow chart from input information to reprogramming determinants detection.** Differential stability analysis takes as input a gene regulatory network and experimental expression data comparing initial and final cellular phenotypes. The output of the analysis consists on combinations of target genes to be perturbed to induce the desired cellular transition.

**Figure 3:**
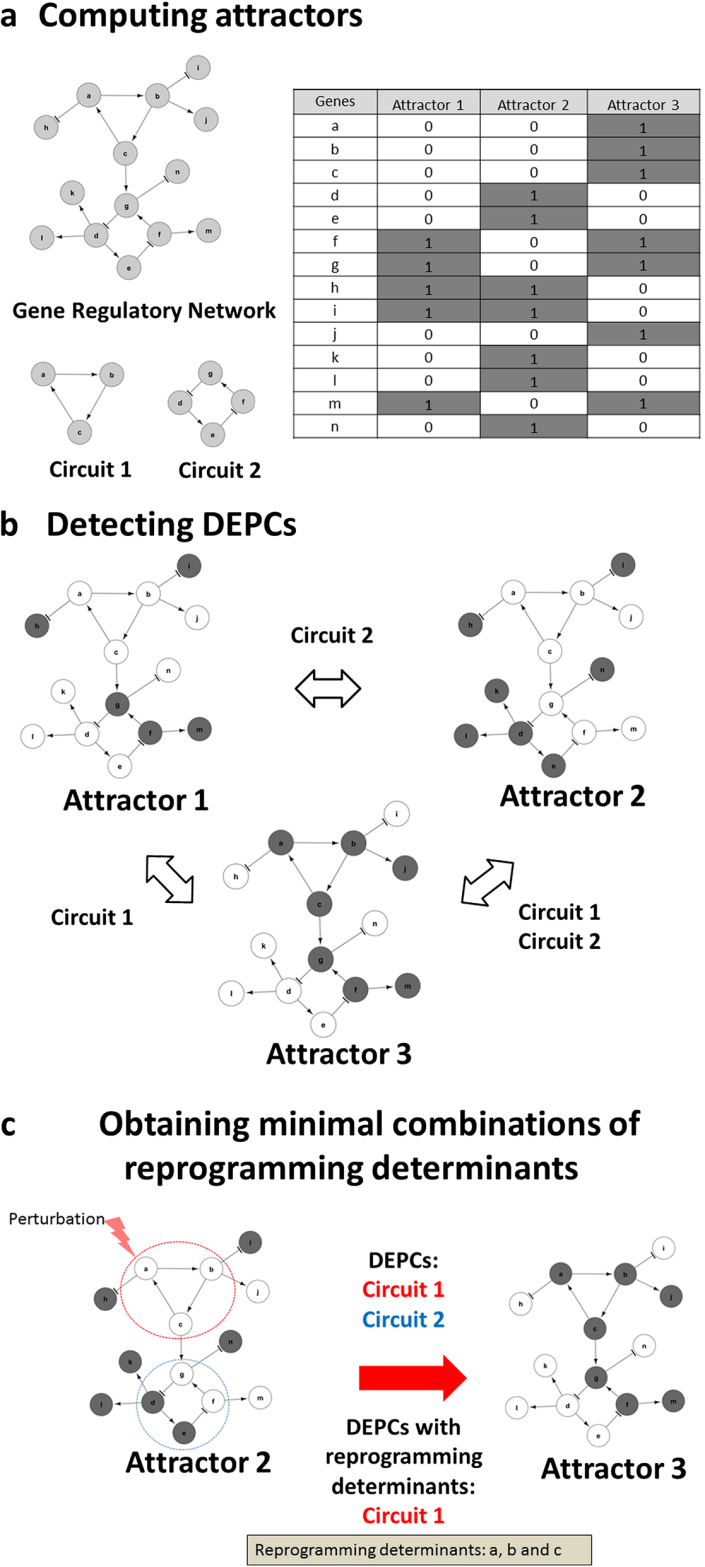
**Differential stability analysis: recipes for cellular reprogramming in three steps. a)** Computing attractors. Network stability is analyzed assuming a Boolean model and a synchronous updating scheme. Genes in “1” are active or “ON” and genes in “0” are inactive or “OFF” and are represented in grey and white respectively. **b)** Detecting DEPCs. A positive circuit is considered a DEPC if all of their constitutive genes change their expression values between two given attractors of the GRN. **c)** Obtaining minimal combinations of reprogramming determinants. Both Circuit 1 and Circuit 2 are DEPCs, but Circuit 2 is regulated by Circuit 1; any perturbation of Circuit 1 capable to move it to a different attractor is going to change the state of Circuit 2 too. Simulations showed that genes in Circuit 2 have not to be perturbed to achieve transition from Attractor 2 to Attractor 3. Therefore, minimal combinations of reprogramming determinants are any individual gene of Circuit 1, i.e., genes “a”, “b” or “c”. Regular arrows represent activation and T-arrows represent inhibitions.

The method takes as input GRNs and experimental expression data and delivers combinations of RDs (see flow-chart in Figure 2) and can be described in three steps (see Figure 3): 1) computing GRN attractors 2) detecting DEPCs 3) obtaining minimal combinations of RDs genes targeting the DEPCs, in detail as follows.

#### Computing attractors of the network

Attractors are calculated with a Boolean model of the GRN (see Methods for details). In this Boolean model, up and down regulated genes assume values of “1” and “0” respectively. This is necessary to find suitable attractors of the network model representing the cellular phenotypes.

#### Detecting DEPCs

At first, all positive circuits are detected using modified Johnson’s algorithm (see Methods section for details). Later, from this set of positive circuits a subset, whose constitutive gene expression profiles are differentially expressed between the attractor states involved in cellular transition (initial and final), are identified. For a positive circuit to be differentially expressed it has to fulfill two requirements: (i) all of their constitutive genes change between the two attractors (i.e., they are differentially expressed), and (ii) the states of the circuit in both initial and final phenotypes should match attractors of the circuit when considered in isolation; (i.e., only circuits in stable state whose logical rules are in accordance with their expression patterns are considered as differentially expressed stability elements).

#### Obtaining minimal combinations of RDs genes targeting all DEPCs

We look for the minimal combination of genes that are able to directly or indirectly target all DEPCs. For this purpose, we formulated this as a two-step integer optimization problem, where in the first step by perturbing all the genes in a given circuit, minimal numbers of circuits that can bring about the cellular transitions are identified. In the second step, minimal combinations of genes are identified from the minimal number of circuits using an algorithm that look for combinations of genes in minimal DEPCs with the requirement that there should be at least one gene for each DEPCs (see Methods). Consequently this strategy reduced further the required number of genes to be perturbed. Afterwards, as a final step, the algorithm determines which DEPCs are not necessary to be directly perturbed (see Figure 3c) by simulating the network response (according to the model assumed to compute attractors) under perturbation of the minimal combination of genes but the gene belonging to specific DEPCs one at a time. By this mean we are able to reduce the final number of RDs removing genes targeting DEPCs that are regulated by others.

### Validation with *in silico* gene regulatory networks

**Figure 4:**
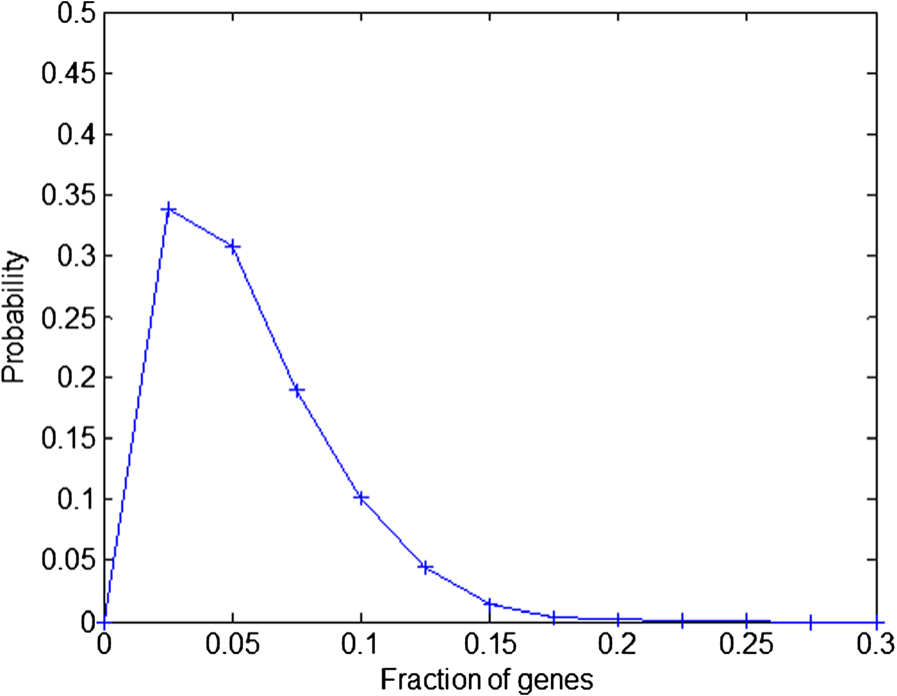
**Probability density function of fraction of genes to be perturbed in the whole network to induce the transitions from any given attractor to any other.** In average, only 6% of the genes in 1000 randomly generated networks preserving E. coli GRN topological properties are identified by our method as RDs.

In order to validate this strategy, we applied our method to 1000 GRNs of different size, but with the same topological properties of a well-characterized GRN of E. coli. As a result of our analysis we obtained the following conclusions: a) Between any two given attractors we always obtained at least one DEPC; and b) perturbation of minimal combinations of genes that include DEPCs between pairs of attractors always succeeded triggering transitions between these states (see Figure 1 as example). Further, we calculated the percentage of RDs that can trigger transitions between all calculated attractors. As it is shown in Figure 4, interestingly on an average only 6% of the genes from the whole network is sufficient enough to bring about the transitions between any given attractor to any other. Also, on an average maximum 4 genes and a minimum of 1 gene is sufficient to bring these transitions.

### Application to cellular reprogramming

We demonstrated the efficacy of the current protocol using six different biological examples of cellular reprogramming. These examples provided an experimental confirmation of the identified RDs as effective inducers of transitions between stable cellular phenotypes. The T-helper and EMT examples are based on GRNs, which have been previously published [15,16]. In the latter case we expanded the original network with the addition of a novel double-negative feed-back with miRNA34A, which has been recently published [17]. For the remaining examples (HL60, iHEP, iCM and iPSc) we used knowledge bases, like Ariadne’s MedScan technology [18,19], to construct gene regulatory networks. We also tried to enrich, when possible, the GRNs by addition of miRNA interactions experimentally validated and available in public databases, like TransmiR [20] and miRTarBase [21] (see Methods for details). Consequently, these networks were pruned in order to maximize matching between gene expression profiles and gene states found by our network dynamics simulation. This procedure allowed us to contextualize the networks to the biological conditions under which the experiments were performed [22] (more details about the network reconstruction and contextualization processes are included in the Methods section below). Detected RDs and transitions between known phenotypes are shown in Table 1 for each example.

**Table 1:** Minimal combinations of reprogramming determinant genes obtained after the application of our method in five different biological examples for specific transition between attractors corresponding to cellular phenotypes

**Example**	**Transitions**	**DEPFCs**	**Minimal combinations of reprogramming determinant genes**
**T-helper**	Th2-Th1	4	GATA3, **T-bet**
**EMT**	Epithelial-Mesenchymal	12	**SNAI1**, ZEB2, MIR203
**HL60**	HL60-Neutrophil	1	IL1B, CASP1, **IRF1**
**iHEP**	Fibroblast-Hepatocyte	2	FOXA2:PPARGC1A,NR5A2:UCP2, HNF1A:PPARGC1A,HNF4A:NR5A2, NR5A2:PPARGC1A, **FOXA2:HNF4A**, HNF1A:UCP2, AGT:NR5A2,AGT:FOXA2,FOXA2:UCP2, AGT:HNF1A, HNF1A:HNF4A
**iCM**	Fibroblast-Cardiomyocyte	2	**GATA4**, MEF2C
**iPSCS**	Fibroblast-iPSCS	7	**MYC:POU5F1:SOX2**, POU5F1:SOX2:mir-107,MYB:POU5F1:SOX2, ATF3:POU5F1:SOX2,POU5F1:SOX2:TP53,

Genes in bold correspond with those whose perturbation are represented in Figure 5.

Alternative combinations of reprogramming determinant genes are separated by comma. Combinations of reprogramming determinants genes perturbed in Figure 6 are in bold.

#### T-helper

T lymphocytes are classified as either T helper cells or T cytotoxic cells. T helper cells take part in cell- and antibody-mediated immune responses and they are sub-divided in Th0 (precursor) and effector Th1, Th2, Th17 and Treg cells. T-helper differentiation network determining the fate of the lineage has been proposed previously [15]. Here we are focused on the transition between Th2 and Th1 phenotypes. We detected T-bet and GATA3 as independent RDs for Th2-Th1 (see Figure 5a) and Th1-Th2 respectively. These predictions are in full agreement with previously published experiments [6,23,24].

**Figure 5:**
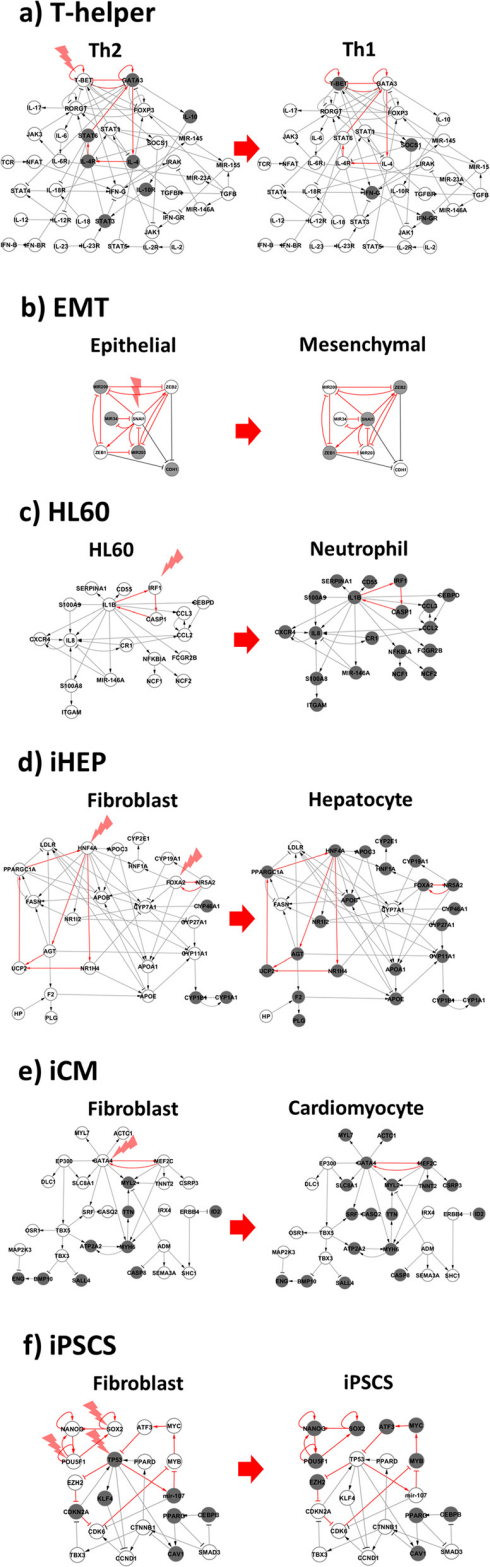
**Six cellular transitions corresponding to six illustrative biological examples are represented in this figure after the perturbation of specific minimal combinations of reprogramming determinant genes.** Simulated perturbations performed assuming a Boolean model succeeded in triggering the transition. These results are consistent with experimental perturbations previously published. Genes in “ON” and “OFF” are represented in grey and white respectively; these states correspond to the characteristic expression profile in both initial and final states. Points of perturbation of DEPCs are marked with red arrow. These genes should change their states in order to induce the desired cellular transition. **a)** T-helper. Perturbation of T-BET induces the transition from Th2 to Th1. **b)** EMT. Perturbation of SNAI1 induces de transition from epithelial to mesenchymal **c)** HL60. Perturbation of IRF1 induces the transition from HL60 to neutrophils. **d)** iHEP. Combined perturbation of HNF4 and FOXA2 induce the transition from fibroblast to hepatocyte. **e)** ICM. Perturbation of GATA4 induces the transition from fibroblast to cardiomyocyte. **f)** iPSCS. Combined perturbation of SOX2, POU5F1 and TP53 induce the transition from fibroblast to iPSCS. Regular and T-arrows represent activation and inhibition respectively.

#### EMT

A transient phenomenon referred to as epithelial to mesenchymal transition (EMT) occurs during regular embryonic development and as a part of the metastatic cascade initiated by the breakdown of epithelial cell homeostasis in carcinomas. During the Epithelial to mesenchymal transition (EMT), cells change their genetic and transcriptomic program leading to phenotypic and functional alterations, including the loss of epithelial features like cell-cell adhesions and cell polarity and gain of cell motility and mesenchymal and stem-like properties. EMT can be initiated by multiple pathways converging in the activation of EMT inducers. The EMT example shows that SNAI1 is a triggering gene for the transition from epithelial to mesenchymal (see Figure 5b), which has been validated by experimental perturbation of this gene [16].

#### HL60

The multipotent promyelocytic leukemia cell line HL60 was originally isolated by Dr. Steven Collins from an acute promyelocytic leukemia (APL) patient [25]. The multipotent promyelocytic leukemia cell line HL60 can be stimulated to differentiate into neutrophils using different chemical agents like including granulocyte macrophage colony-stimulating factor (GM-CSF) [26], DMSO [27], all-trans-retinoic acid (ATRA) [28], 1,25-dihydroxyvitamin D3 [29], and 12-O-tetradecanoylphorbol 13-acetate (TPA) [30]. Nevertheless, how these chemical agents act at the gene regulatory level to induce the transition is still a relevant question to understand the underlying mechanisms of differentiation or reprograming. Application of our method to the HL60 example allowed us to detect IRF1 as triggering gene for inducing the differentiation from HL60 to neutrophil (see Figure 5c), which is a consistent result with previous experimental findings [31].

#### iHEP

Normally, hepatocytes differentiate from hepatic progenitor cells to form the liver during the regular development. However, hepatic programs can also be activated in different cells under particular stimuli or fusion with hepatocytes. The transition from human fibroblasts to hepatocyte-like cells (iHEP) induced by the perturbation of specific combinations of transcription factors has been previously reported by Sekiya and Suzuki [32]. In the iHEP example we found several minimal combinations able to trigger the transition from fibroblast to hepatocyte. Among these minimal combinations, the combined perturbation (activation) of HNF4A and FOXA2 has been experimentally validated [32] (see Figure 5d).

#### iCM

In the postnatal heart during the regular development, a large pool of existing fibroblasts is directly reprogrammed to an alternative fate as cardiomyocytes. No single master regulator of direct cardiac reprogramming has been identified till date, but the combined perturbation of three developmental transcription factors (GATA4, MEF2C and TBX5) has been proposed and validated experimentally as a rapid and efficient way to induce this transition [5]. Our method found that when GATA4 and MEF2C are perturbed separately or in combination (see Figure 5e) are able to trigger the transition from fibroblast to induced cardiomyocyte (iCM), indicating the important role that these genes play in this cellular transition. This finding is partially consistent with the experiment performed by Ieda and Co-workers [5], where GATA4 and METF2C in combination with TBX5 were simultaneously perturbed to achieve this cellular transition. Thus, our results propose the hypothesis that either GATA4 or METF2C are individually capable to trigger this transition. To our knowledge, this prediction has not been experimentally validated in fibroblast-cardiomyocyte transition, but GATA4 has been reported capable to reprogram mesenchymal stromal and P19 cells [33] into cardiomyocytes [34,35].

#### iPSCs

The combined perturbation of POU5F1, SOX2, KLF4 and MYC is known to be effective to induce the reprogramming of human fibroblasts to the iPSCs. We analyzed a previously published microarray dataset [36] of human Fibroblast to iPSCs. Here an initial population of fibroblast is stimulated using above said four Yamanaka transcription factors to induce transitions to iPSCs. The application of our method to this dataset resulted on the identification of POU5F1, SOX2, and MYC as RDs among other alternative combinations. However, KLF4 was not pointed out as RD according to the reconstructed model and by our methodology due to it is not involved in DEPCs. That might be due to missing interactions within the network, one of the limitations of using only interactions from knowledgebase. ATF3, mir-107, MYB and TP53 were detected as suitable alternative targets to accompany POU5F1 and SOX2, being TP53 the only one with a previously reported key role in cellular reprogramming [37]. The blind-folded application of our methodology to the available trancriptomics datasets pointed out well known key genes involved in pluripotency recovery mechanisms and also proposed a handful of alternative candidates.

## Discussion and conclusions

An increasing amount of experimental results showed that only few key driver genes are required for cellular reprogramming. Since stable cellular phenotypes can be considered as attractors of gene regulatory networks, cell fate and cellular reprogramming involve transitions between these attractors. Hence, this implies that by destabilizing the initial attractor and stabilizing the final one, one can induce the required transitions. Here, we present a topology based method to identify minimal set of key genes belonging to specific stability elements (DEPCs), capable to induce transitions between cellular phenotypes when. We call their identification as differential expression stability analysis.

DEPCs detection relies on attractor computation assuming a Boolean model, which is relatively simple and does not require kinetic parameter identification for a given topology. Also, given that we are not interested in a detailed description of the regulatory mechanism we consider a Boolean model suitable for our purposes, but not for the elucidation of transient states. GRN models in this work do not take into account detailed cellular information, such as the strength of regulatory interactions and continuous gene expression values. However, it preserves the regulatory logic that rules the flow of information in gene regulatory networks, and consequently allows to roughly describing stable cellular phenotypes. It is worth mentioning that, given that this methodology neglects important dynamical aspects like kinetic parameters or affinity values, all interactions within the network are equally strong. This simplification may result in incomplete results because we are assuming that perturbing one gene is enough to effectively destabilize the circuit it belongs to, whereas weak interactions in the circuit may interrupt the regulation signal transfer. Such situation would require perturbation of the circuit at different points (genes) and this is something not considered by our approach.

Analysis of a large number of *in silico* gene regulatory networks using a Boolean model showed that for any couple of given attractors there always exists at least one DEPC, and that RDs detected by the application of the methodology presented here were able to trigger transitions between all pairs of attractors. It is worth noting that this detection differs from a previously published work on the dynamical simulations of gene perturbations instead of the former simulations purely topology based approach [11]. Further, we analyzed six different gene regulatory networks that describe representative biological examples (see Figure 4). Namely, we illustrate the process of cellular differentiation (T-helper differentiation and Epithelial-Mesenchymal transitions (EMT)), in which cells lose phenotypic plasticity until they become fully differentiated as well as the remarkable ability of some differentiated cells to be converted into different cell types via cell reprogramming (HL60-Neutrophil, Fibroblast-hepatocyte, Fibroblast-cardiomyocyte and Fibroblast-induced pluripotent stem cell reprogramming). These examples provide an experimental validation of the identified RDs as effective inducers of transitions between cellular phenotypes. Thus, our method can be used to identify RDs that are able to induce transitions between cellular phenotypes, and finding potential applications in the areas of disease modeling, to create novel disease models and in regenerative medicine for formulating new cellular therapies.

The method provides a strategy to induce transitions between cellular phenotypes exploring the stability landscape, eventually with alternative combinations of perturbed genes with the subsequent differences in trajectories. The fact that our methods provides these alternative combinations could help to address three major problems in cell reprogramming: a) Safety in reprogramming process, avoiding undesired turnings often leading into cancer; among the alternative solutions, some combinations of RDs genes inducing risky transitions too close to a tumorigenic profile can be avoided and safe transitions can be selected [38]; b) Efficiency; The reduced set of alternative experimentally testable solutions facilitates finding more efficient strategies to induce cellular transitions; c) Fidelity; The potentially incomplete reprogramming or the appearance of aberrant phenotypes (for instance, no effective equivalence between iPSC and ESC). Such alternative phenotypes could be detected as additional attractors in the stability landscape and can be taken into account to obtain the desired transitions.

It is to note here that the predicted RDs are purely based on the initially assumed GRN from the literature and the finally contextualized GRN topologies (i.e., the connectivity among genes). GRNs in this work are reconstructed fully from literature knowledgebase. Complementarily, a data oriented approach, like co-expression [39] or mutual information [40] based inference techniques, can also be followed for this particular task. Both literature-based and data-based approached have their own advantages and disadvantages. For example, literature-based approach usually integrates interactions described in varying biological contexts, like different cell types, tissues or even organism, hence resulting in noisy GRNs that are not suitable to describe the system. On the other hand, data based approaches require large amount of data to classify statistically the true positive from false positive interactions of GRN, which may arise due to indirect interactions. Considering the data availability (i.e., number of replicates and perturbation studies) in cellular reprogramming, we opt to employ a former approach to contextualize literature-based GRNs with respect to the available data, hence removing noise.

To this end, raw networks reconstructed from literature are contextualized by pruning those interactions that are not consistent with experimentally observed expression data. This contextualization process requires adopting a Boolean dynamical model that is based on a set of assumed regulatory logic functions if specific regulatory mechanisms are not known (see Methods and Additional file 1 for details). Despite the effect of some wrongly assumed regulatory logic functions is partially overcome by the contextualization itself (discussed in the Additional file 1), sometimes both the wrongly assumed regulatory logic rules and/or network incompleteness may lead us to wrong or incomplete set of RD. However, there is a score obtained during the optimization process that represents the percentage of genes that are well explained by the dynamical model for the initial and final cellular phenotype expression profile. This score constitute an indicator of how reliable the predictions (reprogramming determinants) performed on the contextualized network for a given set of regulatory rules are. Leaving apart the first two examples with previously published networks (Thelper and EMT) all the other four examples were reconstructed from the available knowledge in the literature. Genes included in the reconstructed GRNs of the examples are those with experimental evidences of up- or down-regulation, and only the resulting contextualized topology makes them RD, rather than previous reports about their participation in reprogramming events.

Also, the knowledgebase (Patway Studio) used in this work, exploits word association to detect clear sentences, which describe the interactions with regulatory effects. Only these known regulations are included in the database (Mammalian ResNet). For example, the sentences “…we now report that caspase-1-mediated IL-1beta expression in response to…” and “…activation of caspase-1 is required for the efficient production of biologically active IL-1 beta and IL-18…” (both sentences and PubMed identifiers of the original papers are included in the Additional file 1) allow to include the positive interaction between caspase-1 and IL-18. We checked that the interactions included in the reconstructed GRNs were not based on wrong or ambiguous sentences, but we cannot guarantee that they are not the result of the author’s misinterpretation.

As a limitation of this algorithm, the transitions involving cyclic stable states are not yet considered but are subject of possible extension of the method. Modeling transitions between cyclic attractors could be applied to identify driver genes in biological systems with oscillatory behavior. Also, according to the definition of RDs, the output of the present method has exactly one gene for each DEPC, however in general it is possible to perturb multiple genes per circuit that are redundant or even choose those genes that are experimentally feasible to perturb from the identified DEPCs.

Regenerative medicine, where the goal is to replace or regenerate damaged or lost human cells, is a rapidly growing research area [41]. However, current therapies that focus on tissue regeneration are significantly impeded by our limited understanding of how to reprogram cells towards specific cellular populations. Hence cellular reprogramming, including the conversion of one differentiated cell type to another (trans-differentiation) or to a more immature cell (dedifferentiation), has a high relevance for regenerative medicine and disease remodeling [42]. On the other, with ever increasing amount of experimental observations, it is clearly evident that only few key genes, called here as reprogramming determinants (RDs), are more than enough for the orchestration of the complex regulatory events during reprogramming. Although substantial progress has been made in developing experimental reprogramming techniques, to date there is no protocol able to systematically predict RDs that can trigger transitions. In this article, we provide an in-time framework to design protocols to induce transitions between cellular phenotypes providing effective cellular reprogramming (including protocols for differentiation, dedifferentiation, trans-differentiation and pluripotency recovery). This work thus represents a major potential advance in the way we uncover RDs and pathways involved in cellular reprogramming, with enormous scope for regenerative medicine across diverse tissue- and cell-types.

## Methods

### Extraction of *in silico* gene regulatory networks

In order to validate the applicability of our differential expression stability analysis, we tested our algorithm using *in silico* GRNs of known biological properties. To this end, one thousand GRNs of size between 20 and 40 genes were extracted from the E. coli K12 transcriptional network from RegulonDB (http://regulondb.ccg.unam.mx/) using GeneNetWeaver [43] with greedy neighbor selection and including self-regulations. This size range is chosen in accordance with regulatory cores of the selected biological examples. However, potentially it is possible to scale the algorithm for increased size of the regulatory core, but the attractor computation of the network model forms the bottle neck. It is hard to say fixed numbers a priori as the attractor computation relies on both the network size and its complexity.

**Figure 6:**
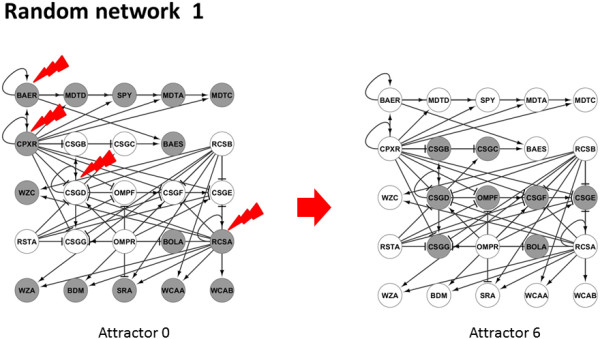
**Example of randomly generated network preserving E. coli topological characteristics.** Perturbation (red pointer) of reprogramming determinants induced the transition from Attractor 0 to Attractor 6. This network includes 25 nodes and 60 interactions. The stability analysis assuming a Boolean dynamical model and a synchronous updating scheme detected 8 stable steady states, so there are 16 possible transitions between them. The transition is achieved after the perturbation of CPXR, CSGD, RCSA and BAER. Such perturbation targets all DEPCs between represented attractors. Genes in “ON” and “OFF” are represented in grey and white respectively. Regular arrows represent activation and T-arrows represent inhibitions.

These *in silico* GRNs, preserves the topological features and the network complexity of the original K12 transcriptional networks (see an example in Figure 6) Since these sub-networks are extracted using preferential node attachment algorithm, the resulting attractor states may or may not represent experimentally observed expression patterns in E. coli. Also, these *in silico* GRNs are only used to portray the effectiveness of our algorithm in a noise free well controlled situation and not to obtain any biological insights, which could have also been achieved by choosing other well studied model organisms like yeast.

### Reconstruction of six biologically relevant examples

We selected six different commonly occurring and biologically relevant reprogramming systems to illustrate the applicability and utility of our method. For the first two examples, T-helper and EMT, we used networks from previously published works [15,16]. For the remaining four examples, i.e., HL60, iHEP, iCM, and iPSc, GRNs were reconstructed from literature using text mining and pathway database tools. Even though, data based inference methods are commonly used to infer the GRNs, to avoid spurious false-positive predictions, we use only experimentally validated regulations from literature. However, potential extension of enriching GRNs with data based network inference methods remains open and out of scope to this work. The main topological properties of the six final networks are shown in Table 2. Details about network interactions and the corresponding attractors of the six examples are included in Additional file 2.

**Table 2:** Number of genes, miRNA interactions and circuits of the six biological examples are shown

**Networks**	**Genes**	**miRNAs**	**Interactions**	**Activations**	**Inhibitions**	**Positive circuits**	**Negative circuits**
**T-helper**	36	4	71	47	24	108	108
**EMT**	4	3	17	2	15	12	0
**HL60**	18	1	30	28	2	2	0
**iHEP**	26	0	57	47	10	12	18
**iCM**	29	0	37	31	6	2	0
**iPSCS**	20	1	34	21	13	9	7

**Figure 7:**
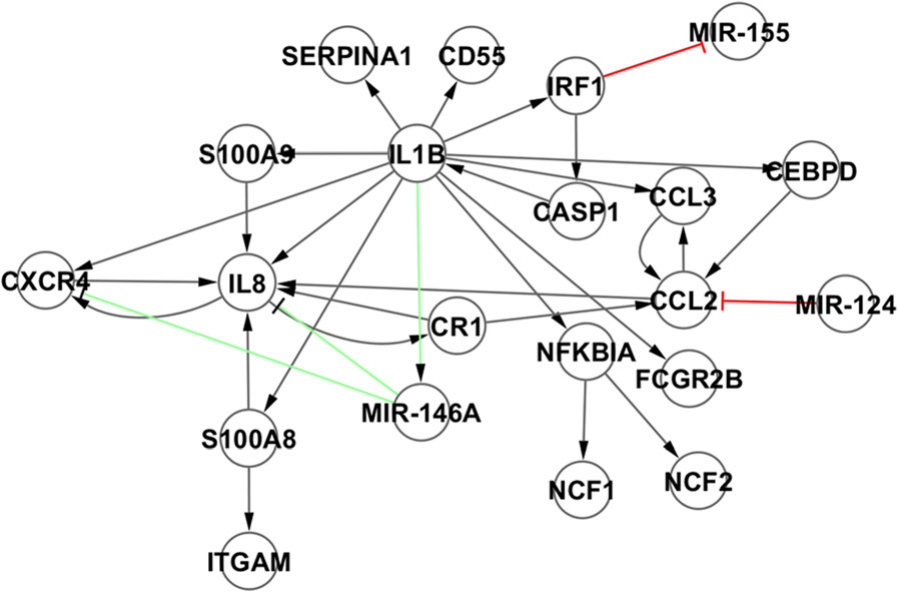
**HL60 GRN.** miRNA interactions included and not included are represented in green and red respectively**.** MIR-146A has incoming and outgoing connections with DEPCs. Both MIR-155 and MIR-124 were removed due to their lack of outgoing and incoming interactions with DEPCS respectively.

The procedure for the network reconstruction consisted of the following steps:

a) *Obtaining a list of differentially expressed genes:* In order to reconstruct GRN, we used set of genes that are differentially expressed between different cell types under consideration. Differentially expressed gene sets for HL60-neutrophil differentiation was obtained from the experiments performed by Mollinedo and co-workers [44]. The fibroblast-hepatocyte and fibroblast-cardiomyocyte gene sets were obtained from the experiments performed by Huang and co-workers [4] and Ieda and co-workers [5] respectively. In the case of the iPSCS example, we analyzed the dataset from the experiments performed by [36]. These sets of differentially expressed genes were obtained after the performance of a *T*-test and selection of genes with a p-value < 0.05.

b) *Inferring regulatory interactions from literature*: GRNs of differentially expressed gene sets were reconstructed using experimentally validated regulation information from literature. For this specific purpose we use the information contained in the ResNet mammalian database from Ariadne Genomics (http://www.ariadnegenomics.com/). The ResNet database includes biological relationships and associations, which have been extracted from the biomedical literature using Ariadne’s MedScan technology [18,19]. MedScan processes sentences from PubMed abstracts and produces a set of regularized logical structures representing the meaning of each sentence. The ResNet mammalian database stores information harvested from the entire PubMed, including over 715,000 relations for 106,139 proteins, 1220 small molecules, 2175 cellular processes and 3930 diseases. The focus of this database is solely on human, mouse and rat. We selected only the interactions included in the ResNet mammalian database in the category of Expression, Promoter Binding, Regulation and Direct Regulation. Interactions in the “Expression” category indicate that the expression of regulatory gene/protein affects their targets, by (both directly and indirectly) regulating its gene expression or protein stability. Interactions in the “Promoter Binding” category indicate that the regulatory gene binds the promoter of the target genes and shows potential regulation experimentally. Interactions in the “Regulation” category indicate that the regulatory gene/protein changes the activity of the target gene/protein indirectly. However, complement to “Regulation” type of interactions, “Direct Regulation” category focuses only on regulations that are effected by means of physical binding. In the inferred interactions, always more preference is allocated to the type interactions which are the result of physical bindings (i.e., Promoter Binding and Direct Regulations). Finally, genes that are not regulated (i.e., nodes without any incoming edges) are iteratively pruned.

c) *Network enrichment with experimentally validated miRNA interactions*: GRNs were enriched, when possible, using miRNA interactions that are publicly available and experimentally validated in two different databases: TransmiR [20] and miRTarBase [21]. These databases potentially include the information about miRNA regulatory genes and miRNA regulated genes, respectively. Since expression data for miRNA’s are not available, miRNA’s forming positive circuits with differentially expressed genes and, therefore, potentially capable of affecting the stability of the network, only were included (see Table 1). However, miRNAs that forms negative feedback circuits and are also potentially participating in system stability are excluded. The reason behind this choice is that the dynamics of such a regulatory motif is not well described in a Boolean representation. In a Boolean system these motifs generate oscillatory behavior, but it is known that in reality this dynamics strongly depends on the kinetic parameters of the interactions [45-47]. We decided not to introduce noise in the model assuming that some regulatory effects could be missing (for example, an increased time response of specific genes under perturbation with the consequent delay in reaching an attractor). On the other hand, a Boolean representation is quite robust to describe stable steady states or fixed points (termed in this paper as attractors) and suitable for our purposes. Information about miRNAs is included in Table 3. Figure 7 shows examples of miRNAs finally not included in the model.

**Table 3:** Interactions with miRNAs included in the examples

**T-helper**	**EMT**	**HL60**	**iHEP**	**iCM**	**iPSCS**
FOXP3 - > MIR-155	MIR200 -| ZEB1	MIR-146A -| CXCR4	None	None	MIR-107 -| CDK6
IFN-G - > MIR-145	MIR200 -| ZEB2	MIR-146A -| IL8			MIR-107 -| MYB
MIR-145 -| STAT1	MIR203 -| SNAI1				TP53 - > MIR-107
MIR-146A -| IRAK	MIR203 -| ZEB2				
MIR-155 -| IFN-GR	MIR34 -| SNAI1				
MIR-155 -| SOCS1	SNAI1 -| MIR200				
MIR-23A -| IL-6R	SNAI1 -| MIR203				
TGFB - > MIR-146A	SNAI1 -| MIR34				
TGFB - > MIR-155	ZEB1 -| MIR200				
TGFB - > MIR-23A	ZEB1 -| MIR203				
	ZEB2 -| MIR200				
	ZEB2 -| MIR203				

The T-helper and EMT examples are based on gene regulatory networks previously published [15,16]. In the latter one we expanded the original network with the addition of a novel double-negative feed-back with recently published miRNA34A [17].

### Attractor computation

Attractor computation was performed assuming a discrete dynamic Boolean model with a synchronous updating scheme [48] (i.e., updates all gene states simultaneously at each step until the system reaches an attractor). Attractors were identified using an in house implementation [22] (written in C++) of the algorithm described by Garg and co-workers, [49]. An inhibitor dominant logic rule was applied to calculate attractors (i.e., if none of its inhibitors and at least one of its activators is active, then a gene becomes active; otherwise the gene is inactive). However, if different regulatory rules are known for specific gene sets, then this knowledge can be included in the model.

### Network contextualization using optimization algorithm

As said earlier GRNs were reconstructed using existing literature, and attractors are calculated for the resulting network model. However, due to the mismatch between resulting attractors and the network interactions, it is necessary to contextualize the reconstructed GRN, which resulted from different experimental conditions, cell types and organisms, to the biological conditions under study. To this end, we applied an evolutionary pruning algorithm [19]. This technique iteratively removes regulatory interactions that are inconsistent between predicted attractor states and experimentally observed expression patterns. Originally, the algorithm was conceived to predict missing expression values in gene regulatory networks, but given all the expression values of a GRN, it could be applied to contextualize the network. The method assumes a Boolean network model to compute its attractors. In gist, this estimation-determination [50] based evolutionary algorithm removes inconsistent interactions, by iteratively sampling the probability distribution of positive circuits and individual interactions within the subpopulation of the best-pruned networks. The resulting contextualized network is based not only on previous knowledge about local connectivity, but also on a global network property (i.e., stability). Given that this contextualization is based on the stability of networks, no assessment can be performed on interactions that are not participating in stability. Due to this fact, in the previous genes that are not regulated by any other genes are iteratively removed.

### Circuit detection

We implemented a modified Johnsons algorithm [51] to detect all elementary circuits, including self-loops in the network. A circuit is a path in which the first and the last nodes are identical. A path is elementary if no node appears twice. A circuit is elementary if no node but the first and the last appears twice. Once we have all elementary circuits, we select positive feedback circuits, or circuits for which the difference between the number of activating edges and the number of inhibiting edges is even. Both elementary circuit detection and positive circuits sorting scripts were implemented in Perl.

### Identification of reprogramming determinants

Once positive circuits are identified, then the differentially expressed sub-set of positive circuits, called as DEPCs are mined. Later, a mixed-integer linear programming formulation is adopted for finding minimum number of DEPCs that can effect cellular transitions. In this formulation, all the genes of a given DEPC or group of DEPCs are perturbed simultaneously and the resulting attractor mismatch with the original attractors are minimized, by varying the combinations of DEPCs to be perturbed.

Once the minimum DEPCs are obtained, then the minimum genes to perturb these minimum circuits, called as RDs, are identified as follows:

1. Detection of the gene represented the most within DEPCs. This gene is added to the growing minimal combination of RDs.

2. Marking DEPCs including this gene as targeted.

3. Checking if there are untargeted DEPCs left. If this is the case, the algorithm goes back to the step 1. If there is no untargeted DEPC left, the algorithm finishes at this point, and the current list of genes constitute minimal combination of genes or RDs.

It is worth mentioning that eventually there are genes drawing in number of targeted circuits. If this is the case the algorithm split the computation in different branches that will provide different alternative RDs.

## Competing interests

The authors declare that they have no competing interests.

## Authors’ contributions

IC conceived the idea, wrote software, carried out the analysis and drafted the manuscript. TMP wrote software, carried out the analysis and drafted the manuscript. WJ carried out the analysis and drafted the manuscript. ADS conceived the idea, drafted the manuscript and supervised the project. All authors read and approved the final manuscript.

## Supplementary Material

Additional file 1 This file includes an explanation based on a small example to illustrate how the network contextualization partially overcomes the problem of assuming wrong regulatory rules or at least provides some guidance about the adequacy of the assumed dynamical model. Click here for file

Additional file 2: Supplementary tables This file includes details about networks for the selected examples and information about computed attractors and the correspondences with cellular phenotypes. Click here for file
