# Supplementary material for: Detecting cellular reprogramming determinants by differential stability analysis of gene regulatory networks
Source: BMC Syst Biol. 2013 Dec 19;7:140. doi: 10.1186/1752-0509-7-140 (PMC3878265; doi:10.1186/1752-0509-7-140)
Supplement: Additional file 1 — This file includes an explanation based on a small example to illustrate how the network contextualization partially overcomes the problem of assuming wrong regulatory rules or at least provides some guidance about the adequacy of the assumed dynamical model. [file 1752-0509-7-140-S1.docx]

**Supplementary Information**

Here, with the help of small gene regulatory networks (GRNs), we illustrate how the network contextualization partially overcomes the problem of assuming wrong regulatory rules or at least provides some guidance about the adequacy of the assumed dynamical model. Also, the characteristic properties of 1000 *in silico* sub-GRNs generated from the GRNs of E. coli K12 from RegulonDB is given.

**S1. Discussion about regulatory rules and network contextualization**

To illustrate the concept, we propose a model GRN with 6 nodes and 7 interactions (see supplementary figure 1). For time being, let us assume that we know the precise regulatory mechanisms for all the genes in the network. This will lead us to create an accurate dynamic Boolean model with logic functions. Hence by using a synchronous updating scheme the attractors can be computed. The most complex regulations in this example are the regulations of ‘A’ by ‘D’ and ‘E’, inhibiting and activating respectively. Given that the effects of these two regulators are the opposite, we have to make a decision about which effect is dominant in case of ‘D’ and ‘E’ are active. Either ‘D’ is dominant and ‘A’ becomes inactive or ‘E’ is dominant and ‘A’ becomes active. In this case, let us assume that the ‘real’ system has the following regulatory rule: if ‘E’ is active and dominant, the state of ‘A’ will be active (irrespective of the state of ‘D’), and if ‘D’ is active and dominant, the state of ‘A’ will be active (irrespective of the state of ‘E’). However, if both ‘E’ and ‘D’ are inactive, let’s assume for time being that ‘A’ will be active. This regulatory logic results in three attractors, whereas if the logic assumed by default is an inhibitory dominant system we only have two attractors (see supplementary table 1).

| 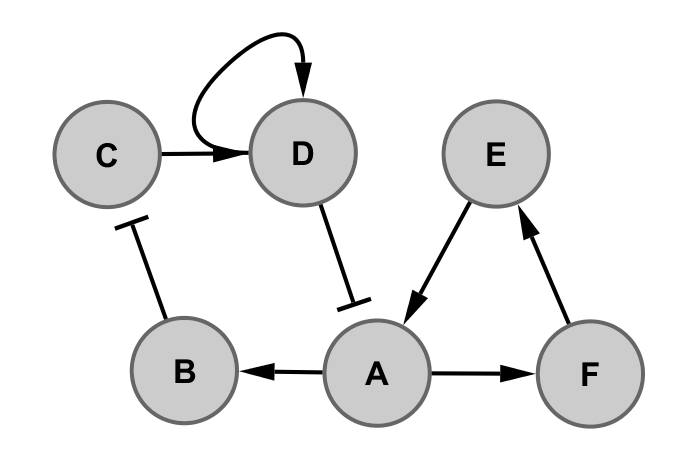 |
| --- |
| **Supplementary figure 1\| Gene regulatory network with 6 genes and seven interactions.** ‘->’ represents activations and ‘-\|’ represents inhibitions. |

Now, let us pretend that we ignore the ‘real’ regulatory rules but we know from experiments that one of the expected phenotypes fits Attractor 3. The problem here is that for the logic rules assumed by default, this state is not an attractor of our system.

| \| **Real regulatory rules** \| **Wrongly assumed regulatory rules** \| \| --- \| --- \| \| **Regulatory rules (logic functions) being the effect of ‘E’ dominant over the effect of ‘D’ when regulating ‘A’** \| **Regulatory rules (logic functions) assuming that a node is active when none of its inhibitors and at least one of its activators is active (1)** \| \| A = E OR (NOT E AND NOT D)  B = A  C = NOT B  D = C OR D  E = F  F = A \| A = NOT D AND E  B = A  C = NOT B  D = C OR D  E = F  F = A \| \| **Resulting attractors** \| **Resulting attractors** \| \| \| Genes \| Attractor 1 \| Attractor 2 \| Attractor 3 \| \| --- \| --- \| --- \| --- \| \| A \| 1 \| 0 \| 1 \| \| B \| 1 \| 0 \| 1 \| \| C \| 0 \| 1 \| 0 \| \| D \| 0 \| 1 \| 1 \| \| E \| 1 \| 0 \| 1 \| \| F \| 1 \| 0 \| 1 \| \| \| Genes \| Attractor 1 \| Attractor 2 \| \| --- \| --- \| --- \| \| A \| 1 \| 0 \| \| B \| 1 \| 0 \| \| C \| 0 \| 1 \| \| D \| 0 \| 1 \| \| E \| 1 \| 0 \| \| F \| 1 \| 0 \| \| |
| --- | --- | --- | --- | --- | --- | --- | --- | --- | --- | --- | --- | --- | --- | --- | --- | --- | --- | --- | --- | --- | --- | --- | --- | --- | --- | --- | --- | --- | --- | --- | --- | --- | --- | --- | --- | --- | --- | --- | --- | --- | --- | --- | --- | --- | --- | --- | --- | --- | --- | --- | --- | --- | --- | --- | --- | --- | --- | --- | --- |
| **Supplementary table 1\| Different regulatory rules can eventually result in different attractors.** The first and second columns refer to real and wrongly assumed regulatory rules respectively in a small toy network. Attractors in a Boolean system using synchronous updating scheme are different between these two cases (Attractor 3 is not an attractor when the regulatory rules are incorrect). |

Now, let us pretend that we ignore the ‘real’ regulatory rules but we know from experiments that one of the expected phenotypes fits Attractor 3. The problem here is that for the logic rules assumed by default, this state is not an attractor of our system. Then we applied the network contextualization procedure. Basically, the algorithm is going to prune the network interactions that make the assumed dynamical model (both topology and logic rules) inconsistent with expression data. In this case, the algorithm is going to remove the interaction ‘D -| A’ (see the resulting logic functions and attractors on supplementary figure 3), whereas when using the real regulatory rules, ‘D -| A’ is perfectly consistent with experimental expression data. Let us see what effect could possibly have these two sets of network topology and logic rules for reprogramming determinants’ detection.

| 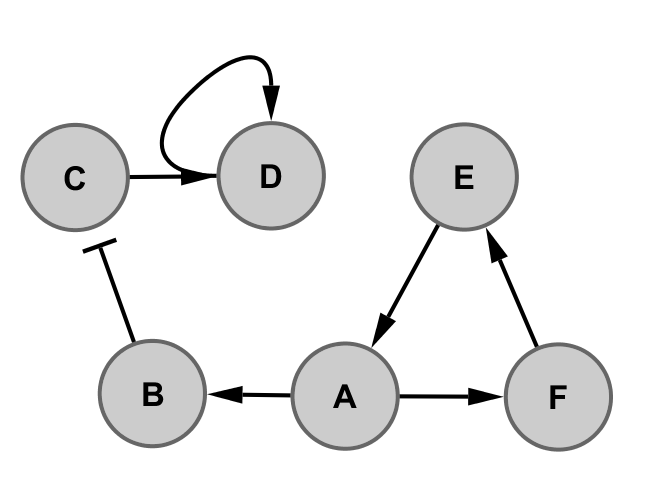 |
| --- |
| \| **Regulatory rules (logic functions) assuming that a node is active when none of its inhibitors and at least one of its activators is in active (1)** \| \| --- \| \| A = E  B = A  C = NOT B  D = C OR D  E = F  F = A \| \| **Resulting attractors** \| \| \| Genes \| Attractor 1 \| Attractor 2 \| Attractor 3 \| \| --- \| --- \| --- \| --- \| \| A \| 1 \| 0 \| 1 \| \| B \| 1 \| 0 \| 1 \| \| C \| 0 \| 1 \| 0 \| \| D \| 0 \| 1 \| 1 \| \| E \| 1 \| 0 \| 1 \| \| F \| 1 \| 0 \| 1 \| \| |
| **Supplementary figure 2\| Contextualized network and the resulting attractors assuming wrong regulatory rules.** ‘->’ represents activations and ‘-\|’ represents inhibitions. |

Let us pretend that we are interested in the transition from Attractor 1 to Attractor 2 and we applied the reprogramming determinants detection procedure in parallel using real and wrong regulatory rules (with the corresponding entire and pruned networks respectively).

In the first case we have three DEPCs (see supplementary figure 3), but in the second one only two because one of the DEPCs has disappeared during the contextualization process (precisely because of the removal of the interaction ‘D -| A’). If we apply the strategy to detect reprogramming determinants to the first case(complete network) we have to select one gene from each DEPC, so we have 1, 3 and 4 possibilities for DEPCs 1, 2 and 3 respectively. When we look for the minimal combination to target all DEPCs we get D:A.

| \| **Real regulatory rules** \| **Wrongly assumed regulatory rules** \| \| --- \| --- \| \| **Contextualized network (no removals)** \| **Contextualized network** \| \| 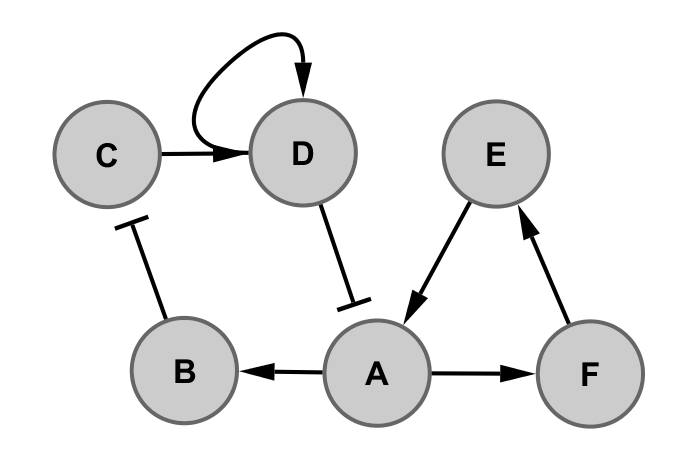 \| 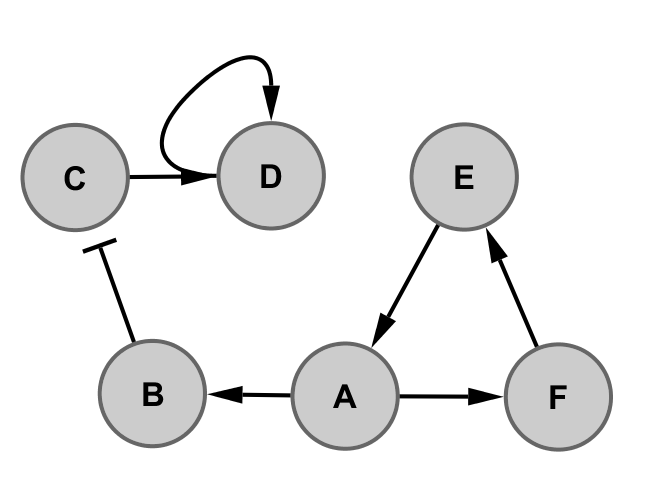 \| \| **Attractor 1 /Attractors 2 DEPCs** \| **Attractor 1 /Attractors 2 DEPCs** \| \| 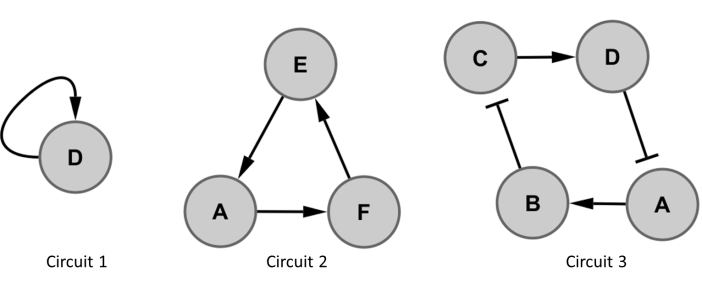 \| 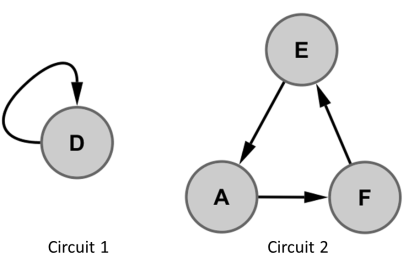 \| \| **Minimal combination of genes to target DEPCs** \| **Minimal combination of genes to target DEPCs** \| \| A:D \| A:D  E:D  F:D \| |
| --- | --- | --- | --- | --- | --- | --- | --- | --- | --- | --- | --- | --- | --- | --- |
| **Supplementary figure 3 \|Finding minimal combinations to target all DEPCs.** The search is performed in both the entire network with the real regulatory rules and the contextualized network (one interaction removed) with the wrongly assumed regulatory rules. In the latter case, Circuit 3 has disappeared, and, given that ‘A’,’E’ and ‘F’ are all of them targeting one positive circuit, they constitute equally valid alternative solutions to target Circuit 2, so the correct combination A:D is among the proposed solutions. |

Now we repeat the procedure in the second model (network without ‘D -| A’). We only have two positive circuits and we have three different minimal combinations D:A, D:E and D:F. Despite we are ‘wrong’ with the selected regulatory rules and the contextualization process forced us to remove a ‘True’ interaction we still get among our combinations or reprogramming determinants the solution we got with the correct topology and regulatory rules. That is possible because despite ‘A’ is not a preferential target anymore (it is as good as ‘E’ or ‘F’), still is an option to target Circuit 2. In other words, breaking circuits because of the wrong selection of regulatory logic rules lead us to more alternative solutions, but still we have the good ones among them.

However, it is not always possible just by pruning (we are not adding or inferring new interactions) to make the dynamical model to explain 100 % of experimental expression values. This statement is valid not only for the case of regulatory rules wrongly assumed but also due to network incompleteness. Nevertheless, during the contextualization process we obtain a score that represents the percentage of genes that are well explained by the dynamical model for the initial and final cellular phenotype expression profile. This score constitutes an indicator of how reliable are the predictions (reprogramming determinants) performed on the contextualized network for a given set of regulatory rules. In addition, it is also possible to run the contextualization using different sets of regulatory rules and check which one gets the best contextualization score. The search for optimal regulatory rules and topology constitutes indeed a natural expansion for the contextualization algorithm that is out of the scope of this work.

It is also worth mentioning that information about new interactions might modify the results (i.e., RDs) if they cause the appearance of new DEPCs within the GRN. There are three possible situations for these new DEPCs:

1. New DEPCs are regulated by old DEPCs. In this case RDs of both original and expanded GRN coincide.
2. New DEPCs are regulating old DEPCs. In this case RDs are going to be different, with new target genes belonging to the new regulating DEPCs instead of in the old DEPCs.
3. New DEPCs are not regulating nor regulated by old DEPCs. In this case the set of genes that constitute the RDs of the original network is incomplete. The RDs of the expanded version of the GRN will require some extra genes to target the new DEPCs.

We admit that it is not possible to know in advance if a given reconstructed GRN is including the DEPCs on the top of a hypothetical hierarchical organization of DEPCs (such a thing would lead us to a situation type a). Still, experimental perturbation of RDs and further characterization of the resulting cell population in a failed transition may help to detect missing information (genes and/or interactions) within the GRN and to guide the experimental research.

**S2. Characteristic properties of the 1000 *in silico* GRNs**

**Supplementary Figure 4| Property distributions of 1000 *in silico* GRNs.** Figures (a)-(f) shows the size and complexity of 1000 gene regulatory sub-networks generated from E. coli K12 regulatory network. (a) Network size, measured as number of genes in the network, (b) Adjacency index, measured as the total number of edges in the network, (c) Average vertex degree, measures as the ration of adjacency index to network size, (d) Connectedness, measured as the ratio of adjacency index to the square of network size, (e) Average path length, measured as the ratio of network diameter to the square of network size, (f) Characteristic path length, measured as the mean short path lengths.
